# Ocular Motor Abnormalities in Anti-IgLON5 Disease

**DOI:** 10.3389/fimmu.2021.753856

**Published:** 2021-09-30

**Authors:** Stefan Macher, Ivan Milenkovic, Tobias Zrzavy, Romana Höftberger, Stefan Seidel, Evelyn Berger-Sieczkowski, Thomas Berger, Paulus S. Rommer, Gerald Wiest

**Affiliations:** ^1^ Department of Neurology, Medical University of Vienna, Vienna, Austria; ^2^ Division of Neuropathology and Neurochemistry, Department of Neurology, Medical University of Vienna, Vienna, Austria

**Keywords:** encephalitis, eye movements, clinical immunology, ocular motor, anti-IgLON5 disease

## Abstract

**Objective:**

Anti-IgLON5 disease forms an interface between neuroinflammation and neurodegeneration and includes clinical phenotypes that are often similar to those of neurodegenerative diseases. An early diagnosis of patients with anti-IgLON5 disease and differentiation from neurodegenerative diseases is necessary and may have therapeutic implications.

**Methods:**

In our small sample size study we investigated oculomotor function as a differentiating factor between anti-IgLON5 disease and neurodegenerative disorders. We examined ocular motor and vestibular function in four patients suffering from anti-IgLON5 disease using video-oculography (VOG) and a computer-controlled rotational chair system (sampling rate 60 Hz) and compared the data with those from ten age-matched patients suffering from progressive supranuclear palsy (PSP) and healthy controls (CON).

**Results:**

Patients suffering from anti-IgLON5 disease differed from PSP most strikingly in terms of saccade velocity and accuracy, the presence of square wave jerks (SWJ) (anti-IgLON5 0/4 *vs*. PSP 9/10) and the clinical finding of supranuclear gaze palsy (anti-IgLON5 1/4). The presence of nystagmus, analysis of smooth pursuit eye movements, VOR and VOR suppression was reliable to differentiate between the two disease entities. Clear differences in all parameters, although not always significant, were found between all patients and CON.

**Discussion:**

We conclude that the use of VOG as a tool for clinical neurophysiological assessment can be helpful in differentiating between patients with PSP and patients with anti-IgLON5 disease. VOG could have particular value in patients with suspected PSP and lack of typical Parkinson’s characteristics. future trials are indispensable to assess the potential of oculomotor function as a biomarker in anti-IgLON5 disease.

## Introduction

The clinical phenotype of anti-IgLON5 disease mainly comprises features of a neurodegenerative disease. Patients diagnosed with anti-IgLON5 disease appear to have four core syndromes: 1) sleep disorder 2) bulbar dysfunction 3) progressive supranuclear palsy (PSP) - like syndrome 4) cognitive impairment (1). IgLON5 is a cell adhesion molecule belonging to the immunoglobulin superfamily. Its proteolytic cleavage from the cell surface plays an important role in neurite outgrowth, although its exact function is poorly understood (2, 3). Neuronal dysfunction and finally neurodegeneration is assumably triggered by the binding of anti-IgLON5 antibodies to its epitope, leading to hyperphosphorylation and accumulation of tau protein. The etiological role of inflammation is so far controversial. Autopsy studies have shown the absence of inflammatory infiltrates, but subcortical neuronal accumulation of tau isoforms 3repeat (3R) and 4repeat (4R) with predominant involvement of the hypothalamus and the brainstem (4). Recently, however, no phosphor-tau (pTau) deposits were found in a patient with relatively rapid disease progression, but CD8+ T cell infiltrates were seen (5). An immunological component may therefore be discussed in early disease stages. The neuroinflammatory hypothesis of PSP suggests microglia activation even in early stages of the disease and expression of proinflammatory cytokines, linking neuroinflammation with neurodegeneration, thus contributing to the pathogenesis of PSP (6).

Patients suffering from PSP mainly show four core symptoms: 1) ocular motor dysfunction consisting of vertical supranuclear gaze palsy, slow velocity of vertical saccades or square wave jerks, 2) postural instability, 3) akinesia, and 4) cognitive dysfunction (7). Other symptoms include dysphagia and dysarthria. PSP-like syndrome was defined as a hallmark of anti-IgLON5 disease (1). Anti-IgLON5 disease usually differs from PSP in typical sleep disorders, although one in two patients has supranuclear gaze palsy or other symptoms that mimic atypical Parkinson’s syndrome (1, 8–10). A previous study showed that the majority of patients with anti-IgLON5 disease had parasomnia or sleep disturbances but also brainstem involvement with dysarthria, dysphagia, vocal cord paralysis, sialorrhea, respiratory failure or stridor (1, 11). On the other hand, patients suffering from PSP do not have anti-IgLON5 antibodies (11). Assuming that immunological mechanisms trigger neurodegenerative disease, immunotherapy may only be effective early in the course of the disease. The irreversibility of antibody-mediated internalization of the antigen and the uncertain role of inflammatory changes (especially in patients with long disease duration) may also contribute to poor prognosis and little treatment effect. Early differentiation from PSP patients therefore seems urgently necessary in order to start suitable therapy regimens as soon as possible. Saccadic smooth pursuit and mild horizontal gaze-evoked nystagmus, hypometric vertical and horizontal saccades, supranuclear gaze palsy, horizontal gaze palsy and ptosis have been reported in anti-IGLON5 disease (12–14). Eye movement disorders in anti-IgLON5 disease have been recorded with polysomnography (PSG), but a quantitative characterization of the ocular-motor phenotype by video-oculography (VOG) has not yet been reported.

Saccades are fast eye movements that redirect the eyes onto an object that attracts attention in order to show it sharply in the fovea. Voluntary saccades usually show latencies of about 200ms and large saccades may have peak velocities beyond 500°/sec. In a simplified model horizontal and vertical eye movements are controlled by the paramedian pontine reticular formation and the rostral interstitial nucleus located in the midbrain respectively. Besides these two centers the superior colliculus, the frontal eye field and the cerebellum are the main structures involved in initiating, targeting and controlling saccadic eye movements. Smooth pursuit eye movements are necessary to continuously hold moving objects steady upon the fovea. Retinal ganglion cells project *via* the lateral geniculate nucleus to the striate cortex and extrastriate areas. Further main neural areas involved in smooth pursuit eye movements are the frontal eye field, the supplemental eye field, the pontine nuclei and the cerebellum. In normal subjects the VOR moves the eyes at the same speed but in the opposite direction to the head movement which is accomplished by semicircular canals projecting to the vestibular nuclei and ocular motor nuclei (15).

In this small cohort we aimed to explore differences in ocular motor function between patients with anti-IGLON5 disease and PSP patients in order to support early diagnosis of anti-IGLON5 disease. The primary objective was to characterize horizontal and vertical saccadic abnormalities in the above-mentioned diseases in comparison to healthy controls (CON). Further, we highlight differences in smooth pursuit eye movements (SPEM), vestibulo-ocular reflex (VOR) function and VOR suppression.

## Methods

We investigated quantitative VOG data of patients with anti-IgLON5 disease and compared them with those of patients with progressive supranuclear palsy-parkinsonism (PSP-P) or classic Richardson’s syndrome (PSP-RS) and CON with normal ocular motor function (see below). VOG data of patients diagnosed with anti-IgLON5 disease between years 2015 and 2018 and of patients with PSP, diagnosed between years 2012 and 2018 have been reviewed and analyzed retrospectively.

### Subjects

Four patients suffering from anti-IgLON5 disease and ten patients diagnosed with PSP (5 PSP-RS, 5 PSP-P) underwent VOG and rotational chair testing. Patients were diagnosed according to the established 2017 International Parkinson’s and Movement Disorder Society (MDS) Criteria for the Diagnosis of PSP (7). Patients enrolled before 2017 were revisited and classified according to the recent MDS-PSP criteria for PSP-P and PSP-RS. Diagnosis of anti-IgLON5 disease was supported by the use of established criteria for autoimmune-encephalitis and confirmed by anti-IgLON5 antibody detection (1, 14, 16). Auto-antibodies were determined using a well-established in-house routine cell-based-assay as reported earlier (17). No other antibodies were found in patients with anti-IgLON5 disease, and no antibodies (including anti-IgLON5 antibodies) were found in patients with PSP. All patients underwent VOG at least once, the VOG-recording closest to time of diagnosis was used for analysis. Patients were excluded from VOG if any restrictions were present that would have limited the patient’s cooperation during the examination. The protocol was approved by the local ethics committee of the Medical University of Vienna (EK nr. EK 1773/2016; 1123/2015).

### Controls

Ten sex- and age-matched subjects without history of any neurological disease and with normal neurological examination served as CON.

### Video-Oculography and Rotational Chair System

VOG and a computer-controlled rotational chair system (System 2000, Micromedical Technologies, Illinois, USA), were used to assess ocular motor and vestibular function (sampling rate 60 Hz). The patient’s head was fixed in a u-shaped headrest at suboccipital height. Stimuli were applied by a laser projector.

### Protocol

#### Spontaneous Eye Movements

In this task the patients were instructed to fix an imaginary point on the wall for 30 seconds in complete darkness while looking straight ahead and not to move their eyes.

#### Saccades and Square Wave Jerks

For horizontal and vertical saccade testing, the target moved randomly in amplitude steps of 5° - 30° in both directions in the horizontal and vertical plane, respectively. Saccades were detected by velocity thresholding algorithms which classified points as a saccade if their velocity was greater than a threshold, and as a fixation otherwise. A 100% saccade accuracy was achieved if the patient hit the target exactly. Any value below or above 100% was considered as undershoot (hypometria) or overshoot (hypermetria) respectively. SWJ during fixation and straight-ahead gaze in the dark were visually evaluated by a specialist after recording and subsequently their frequency was determined after manual correction of artifacts.

#### Smooth Pursuit Eye Movement

We investigated SPEM at 0.1, 0.2 and 0.4 Hz. Patients were instructed to follow a sinusoidally moving target in the horizontal plane with a maximum amplitude of +/- 15°.

#### Vestibulo-Ocular Reflex, VOR Suppression

The VOR was tested in complete darkness at 0.32 Hz (chair velocity 30 dps). VORs was tested at 0.04 Hz (chair velocity 60 dps).

The analysis of eye movements was performed automatically by system-specific analysis algorithms (Micromedical Technologies). All recordings were visually cross-checked and artefacts were manually removed by experienced specialists.

### Statistical Analysis

The statistical analyses were performed using IBM SPSS Version 26.0. For parametric correlations, Pearson correlation coefficients were used, and for non-parametric correlations, Spearman´s rho was calculated. Horizontal and vertical saccades were divided into two groups with respect to their amplitude 1) 0-15° and 2) 15-30°. Inter-group comparisons were calculated using Kruskal-Wallis test. The significance level was set at 0.05 adjusted with Bonferroni correction for multiple testing. The values were given as median, interquartile range (IQR) and minimum to maximum (min.-max.).

## Results

Clinical signs and findings (see **Table 1**):

**Table 1:** Baseline characteristics at time of diagnosis and symptoms at last follow-up visit.

ID	Diagnosis	Antibodies	Age at diagnosis, sex	Time first symptoms to diagnosis/VOG	Initial symptoms	Symptoms at last follow-up (FU) visit					cMRI	Tumor	mRS
						Sleep disorder	Movement disorder	Cognition	Bulbar dysfunction	Other		y/n	Last FU
Pat.1	Anti-IgLON5 disease	S 1:400 C 1:256	64, f	2 /5 years	cognitive dysfunction, epileptic seizures	#, +	–	+	–	da, pnp	ha (b)	n	2
Pat. 2	Anti-IgLON5 disease	S 1:3200 C 1:16	72, f	5 /5 years	stridor, vocal cord palsy	#, +	+	+	+	da	le	n	4
Pat. 3	Anti-IgLON5 disease	S 1:12.800 C 1:128	77, f	10 /10 years	vertigo, gait unsteadiness	#, +	+	+	+	pnp	le	y	6
Pat. 4	Anti-IgLON5 disease	No titer available	unknown, m	14 (post mortem) /8 years	dysphagia, ataxia	#, +	+	+	+	pnp	iat	n	6
Pat. 5	PSP-RS	No	70, m	2 /2 years	dysarthria, falls, gait unsteadiness cognitive dysfunction, aphasia, tremor	#, +	+	+	+	da	le	n	6
Pat. 6	PSP-RS	No	68, m	8 / 10 years	gait unsteadiness, dysarthria, bradykinesia	*-	+	-	+	pnp	n.a.	n	4
Pat. 7	PSP-RS	No	74, m	3 /5 years	falls, bradykinesia, gait unsteadiness, rigor, dysarthria, dysphagia, extensive snoring	*+	+	-	+	-	ga	n	4
Pat. 8	PSP-RS	No	73, f	1 /1 year	falls, gait unsteadiness, dysarthria, dysphagia	*-	+	-	+	-	le	n.	6
Pat. 9	PSP-RS	No	67, f	2 /2 years	limb ataxia, gait unsteadiness, dysarthria, apraxia	*-	+	-	+	-	ca, ma	n	6
Pat. 10	PSP-P	No	66, f	2.5 / 3 years	dysphagia, dysarthria, gait unsteadiness, falls, cognitive dysfunction	*-	+	+	+	-	ga, ma, cs, ha	n	4
Pat. 11	PSP-P	No	70, f	2 / 4 years	dysphagia, dysarthria, gait unsteadiness	*, +	+	+	+	-	ga	n	5
Pat. 12	PSP-P	No	65, m	1.5 / 2 years	rigor, gait unsteadiness	*, -	+	-	+	-	le	n	4
Pat. 13	PSP-P	No	68, f	1.5 / 2 years	supranuclear gaze palsy, gait unsteadiness	*, -	+	-	+	-	ta (b)	n	4
Pat. 14	PSP-P	No	72, m	1.5 / 2 years	gait unsteadiness, falls, bradykinesia	*, -	+	+	-	-	le	n	3

Sleep disorder—confirmed by: video PSG #, by history*; movement disorder: parkinsonism, chorea, facial spasm, myoclonus, ataxia; bulbar dysfunction: dysphagia, dysarthria, stridor, vocal chord palsy, sialorrhea, respiratory failure; other: da, dysautonomia (excessive sweating, bradycardia/tachycardia), pnp, polyneuropathy; MRI: ha, hippocampal atrophy (u, unilateral; b, bilateral), ta- temporal atrophy (u, unilateral; b, bilateral), ca, cerebellar atrophy; ma, mesencephalic atrophy; cs, colibri sign; ga, generalized atrophy; le, leukoencephalopathy; iat, ischemic area thalamus; S, serum; C, cerebrospinal fluid
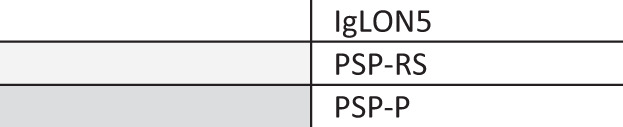

### Anti-IgLON5 Disease

At the time of occurrence of first symptoms, patients with anti-IgLON5 disease had a median age of 66.5 years (62-67 years, min.-max.), the time from onset of symptoms to diagnosis was median 5 years (2-8 years, min.-max.) in three patients. Initial studies with cerebrospinal fluid (CSF) showed normal cell counts in three patients and mild pleocytosis (7 c/µl) in one patient, as well as some slight increase in total protein (42.6 – 54.2 mg/dl; normal range 20 – 40mg/dl) in all patients. No blood-brain-barrier disruption was found. Intrathecal immunoglobulin (Ig) production was not detected, oligoclonal bands (OCB) were negative in all patients. Nerve conduction velocity studies showed evidence of sensorimotor polyneuropathy in three patients with anti-IglON5 disease at the time of diagnosis (patient 1, 3, 4). Three patients received first-line immunotherapy (pulsed high dose corticosteroids, intravenous immunoglobulins, plasmapheresis) and two patients received at least one second-line immunotherapy (azathioprine, rituximab, cyclophosphamide). Under continuous immunotherapy, those two patients stabilized over five and two years respectively. The median time interval between the onset of symptoms and the performance of VOG was 7 years (4.5 – 10 years, min.-max.) In patient 3, tumor screening revealed an intraductal papillary mucinous neoplasm of the pancreas, which we did not consider as causal in view of the long course of her symptoms.

### Progressive Supranuclear Palsy

All patients met the diagnostic criteria for probable PSP. The diagnosis and predominance type were defined by movement disorder specialists. The median age of the patients was 69 years (min.-max. 65-74 years), the median time from first symptoms to diagnosis was 2 years (min.-max. 1-8 years). The median time from initial symptoms to VOG-examination was 2 years (min.-max. 1-10 years). CSF was available from 3 patients and showed increased total protein in all cases, and intrathecal IgG production in one patient. Serum and, if available, CSF antineuronal surface and intracellular antibodies were negative in all cases. At the last follow-up, all patients with PSP showed at least two core symptoms of anti-IgLON5 disease, four patients showed at least three key symptoms of the disease. Nerve conduction velocity studies were conducted upon clinical indication only and revealed polyneuropathy in a single patient.

### Saccade Accuracy

Patients with anti-IgLON5 disease showed significantly higher saccade accuracy compared to patients with PSP for all large and small horizontal and vertical saccades. CON showed significantly higher saccade accuracy for all types of saccades compared to patients with PSP. In patients with anti-IgLON5 disease, saccade accuracy did not differ significantly from CON, except for the accuracy of large horizontal saccades. There was no significant difference in saccade accuracy between patients with PSP, details see **Supplement Table 1**.

### Saccade Velocity

**Figure 1:**
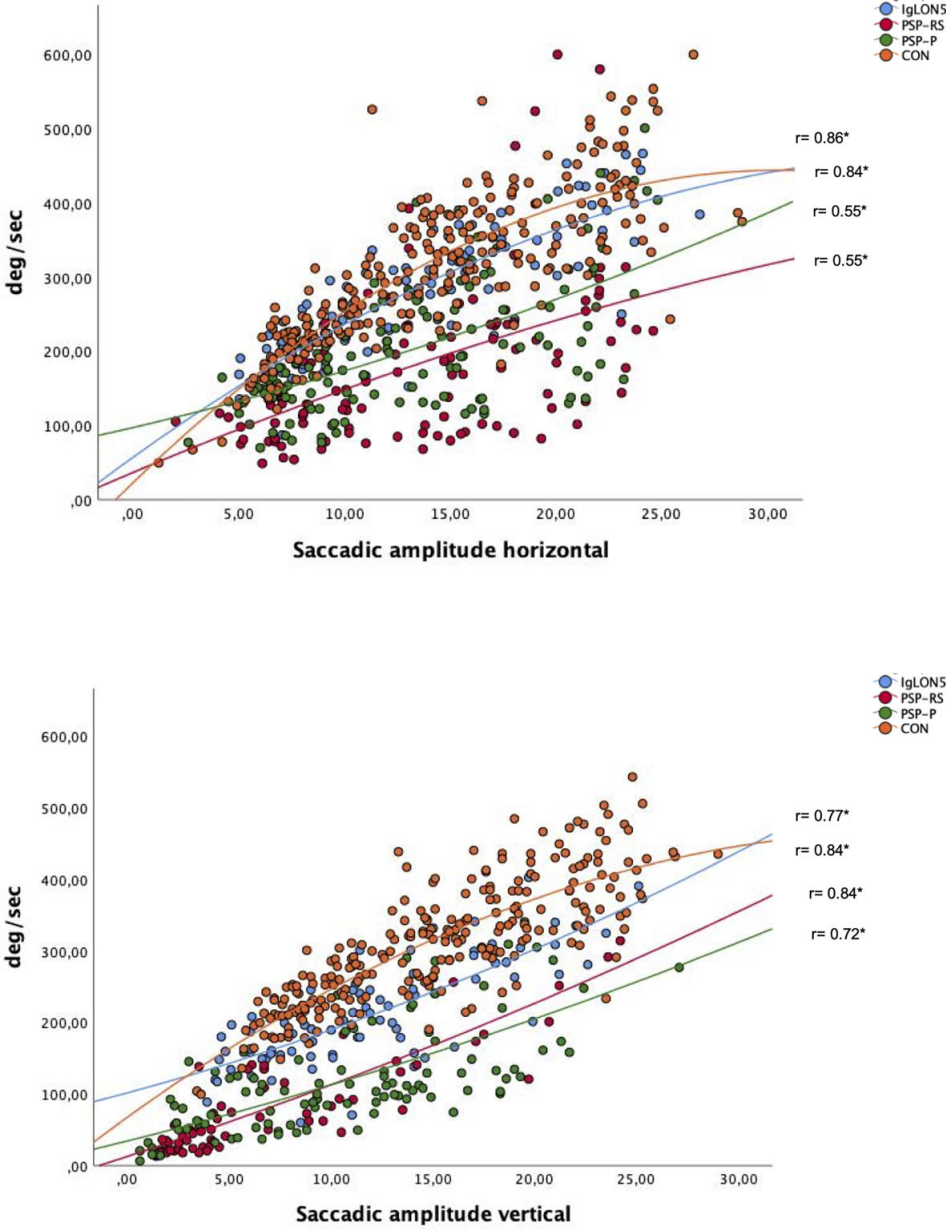
Plot of relationship between saccade amplitude and velocity for horizontal and vertical saccades; significant results p= <0.01 are marked with *. Spearman’s rho is noted in the blot.

Patients with anti-IgLON5 disease showed a significantly higher saccade peak velocity for all types of saccades compared to patients with PSP, except for the velocity of large vertical saccades. CON showed significantly higher saccade peak velocity in all types of saccades compared to patients suffering from PSP. Compared to patients with anti-IgLON5 disease, CON showed significantly higher peak velocities in small and large vertical saccades. Patients with PSP-P did not differ from patients with PSP-RS concerning saccade peak velocity. For details please refer to **Supplement Table 2**. We noted a physiological plateau phase for horizontal and vertical saccade velocity at higher amplitudes in the control group and for horizontal saccades in the anti-IgLON5 group as well as significant differences between patients suffering from PSP and the other groups (see **Figure 1**).

### Saccade Latency

Patients suffering from anti-IgLON5 disease showed significantly lower saccade latency for large horizontal saccades compared to patients with PSP-RS and small and large vertical saccades compared to patients with PSP. CON showed significantly lower saccade latency in all saccade types compared to patients with PSP and anti-IgLON5 disease except for small horizontal saccades. There was no significant difference in saccade latency between patients with PSP, see **Supplement Table 3** for details.

**Figure 2:**
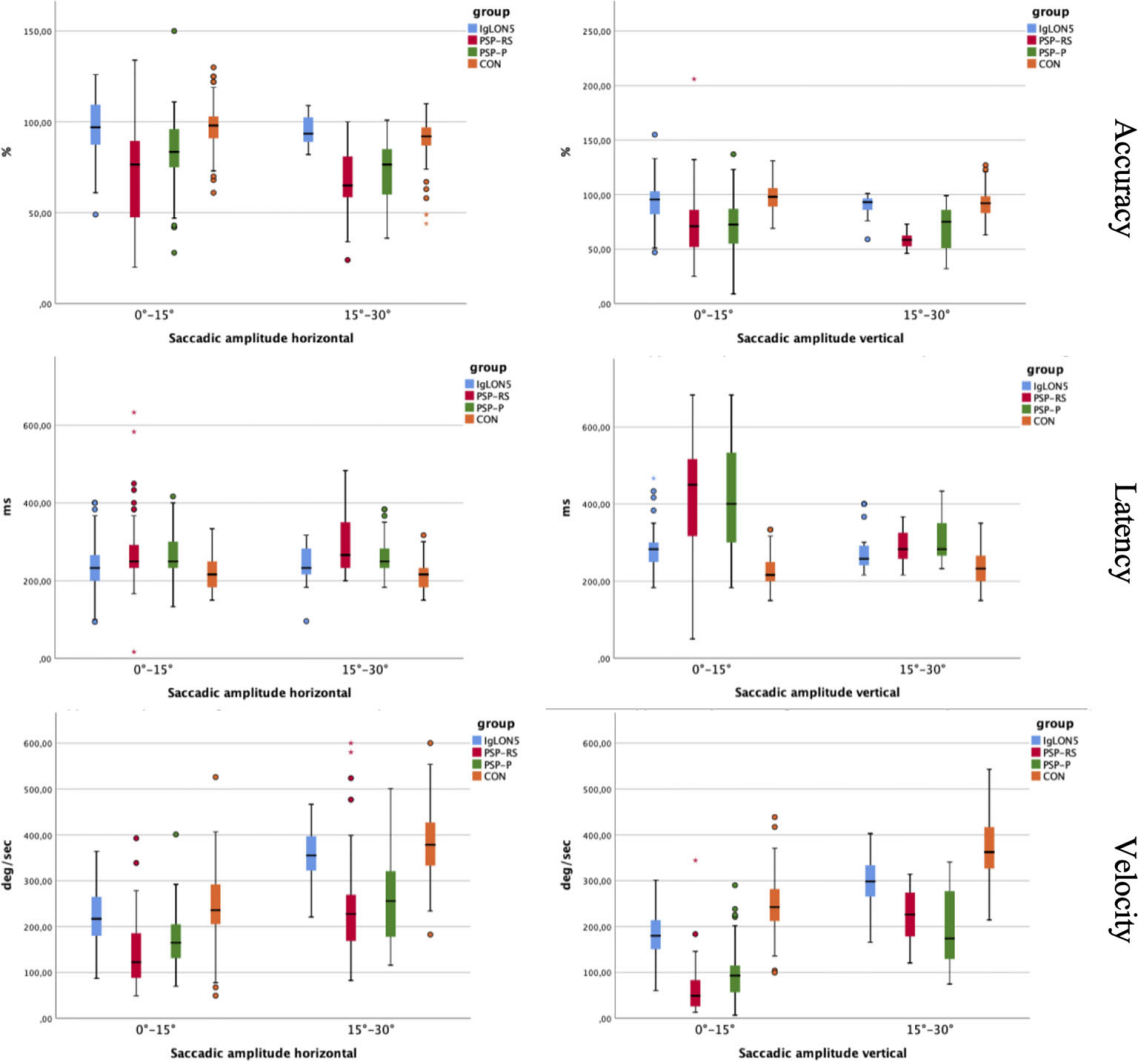
Shows saccade accuracy, velocity and latency of small and large saccades between the groups. Asterisks indicate extreme outliers that have more than 2.5 times the interquartile range from the third or first quartile.

For overview of saccade parameters in detail please refer to **Figure 2**.

### Nystagmus

Patients with anti-IgLON5 disease presented with horizontal spontaneous nystagmus (2/4), upbeat nystagmus (1/4) and bidirectional gaze evoked nystagmus (1/4). In patients suffering from PSP we observed horizontal spontaneous nystagmus (2/10), downbeat nystagmus (2/10) and bidirectional gaze evoked nystagmus (1/10) (see **Table 2**).

**Table 2:** Additional ocular motor and vestibular findings.

Pat.	Nystagmus (slow phase velocity, deg/s)	Fixation	SWJ	Smooth Pursuit gain (0.1 Hz)	Smooth Pursuit gain (0.2 Hz)	Smooth Pursuit gain (0.4 Hz)	VOR gain(0.32 Hz)	VORa (%)	VORp (deg)	VORs(gain)
1	Upbeat (1)	Supp.	No	0.76	0.67	0.61	0.90	0	1	0.32
2	SPN left (1)	Supp.	No	0.87	0.61	0.84	0.89	0	16	0.09
3	SPN right (1)	Supp.	No	0.74	0.79	0.74	0.83	2	24	0.34
4	GEN (h, b)	n.a.	No	0.26	0.22	0.14	0.17	9	41	n.a.
5	No	n.a.	Yes	0.39	0.36	0.46	0.57	4	14	0.19
6	SPN right (1)	Supp.	No	0.84	0.80	0.83	0.51	10	11	0.12
7	DBN (4)	Supp.	Yes	0.49	0.51	0.58	0.76	4.5	14	0.31
8	No	n.a.	Yes	0.78	0.73	0.71	0.77	1.5	3.5	0.23
9	No	n.a.	Yes	0.52	0.31	0.33	0.88	2.5	2.5	0.40
10	GEN (h, b)	Supp.	Yes	0.67	0.55	0.47	0.9	0	27	0.29
11	DBN	Supp.	Yes	0.73	0.74	0.74	0.81	11	6	0.31
12	SPN left (1)	Supp.	Yes	0.76	0.88	0.81	0.74	4	2	0.13
13	No	n.a.	Yes	0.83	0.67	0.63	0.90	0	4	0.43
14	No	n.a.	Yes	0.86	0.86	0.84	0.66	0.4	1	0.1

Sleep disorder—confirmed by: video PSG #, by history*; movement disorder: parkinsonism, chorea, facial spasm, myoclonus, ataxia; bulbar dysfunction: dysphagia, dysarthria, stridor, vocal chord palsy, sialorrhea, respiratory failure; other: da, dysautonomia (excessive sweating, bradycardia/tachycardia), pnp, polyneuropathy; MRI: ha, hippocampal atrophy (u, unilateral; b, bilateral), ta- temporal atrophy (u, unilateral; b, bilateral), ca, cerebellar atrophy; ma, mesencephalic atrophy; cs, colibri sign; ga, generalized atrophy; le, leukoencephalopathy; iat, ischemic area thalamus; S, serum; C, cerebrospinal fluid.
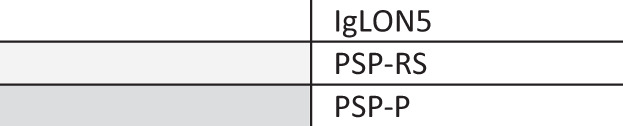

### Saccadic Intrusions

Nine patients suffering from PSP presented with square wave jerks (SWJ). Two patients presented with SWJ only in complete darkness, five patients only during fixation and two other patients presented with SWJ in both conditions. No SWJ were observed in patients with anti-IgLON5 disease, detail see **Table 2**.

### Smooth Pursuit Eye Movements

SPEM was saccadic in all patients, but not in CON. We did not find any significant differences in terms of smooth pursuit gain, comparing results of patients with anti-IgLON5 disease, PSP-P and PSP-RS at 0.1 Hz, 0.2 Hz and 0.4 Hz.

**Figure 3:**
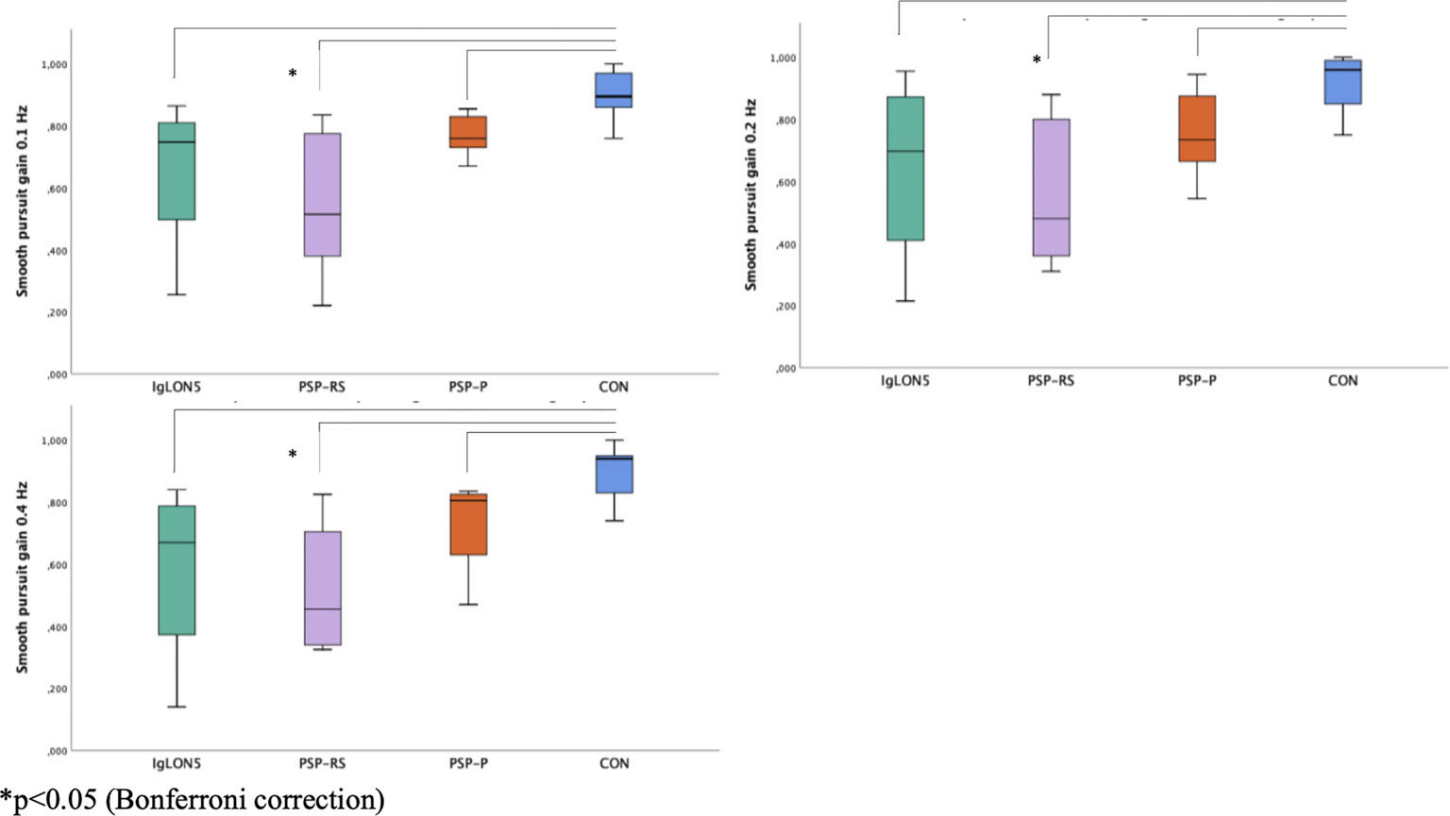
Smooth pursuit gain at 0.1 Hz, 0.2 Hz, 0.4 Hz.

CON (0.90, IQR 0.14, min.-max. 0.76-1.00) showed significantly better performance of smooth pursuit than patients with PSP-RS (p=0.02) but not compared to anti-IgLON5 disease (p=0.11) or PSP-P (p= 0.15) at 0.1 Hz. At 0.2 Hz and 0.4 Hz we observed significant better performance of CON compared to patients with PSP-RS (0.2 Hz p=0.03 and 0.4 Hz p= 0.01) but not compared to IgLON5 (0.2 Hz p= 0.21, 0.4 Hz p= 0.10) and PSP-P (0.2 Hz and 0.4 Hz p= 0.30), see **Table 2** and **Figure 3**.

### Additional Vestibular Findings

#### Sinusoidal VOR/VOR Suppression

We could not detect significant differences in sinusoidal VOR-gain at 0.32 Hz, VOR- asymmetry or VOR-phase between all groups. Compared to CON (0.09, IQR 0.08, min.-max. 0.04-0.23) VORs was significantly reduced in patients with PSP (PSP-RS 0.28, IQR 0.20, min.-max. 0.12-0.40, p= 0.01; PSP-P 0.29, IQR 0.33, min.-max. 0.10-0.43, p= 0.03) but narrowly not significant compared to patients with anti-IgLON5 disease (0.32, min.-max. 0.09-0.34, p= 0.06) (see **Supplementary Figure 1**).

## Discussion

Abnormal eye movements in patients with anti-IgLON5 disease have been described in various studies (1, 8, 14, 18). The novelty of our work is that ocular motor parameters were examined for the first time in anti-IgLON5 disease using VOG and compared with data from neurodegenerative diseases and CON. PSP patients were selected for our analyses because of the clinically similar phenotype as in anti-IgLON5 disease.

The most important results of our study are: I) Determination of saccade accuracy, velocity and latency yielded the most distinct results between patients with anti IgLON5 disease and PSP but also in order to distinguish both entities from healthy controls. Differences between patients with anti-IgLON5 disease and PSP were smaller compared to the difference between PSP and CON. The most obvious differences between patients with anti-IgLON5 disease and PSP were observed for saccade accuracy and velocity, as well as latency. II) Square wave jerks are saccadic intrusions which were more likely to occur in patients suffering from PSP but not anti-IgLON5 disease in our cohort. III) Gain of SPEM was significantly lower in patients with PSP-RS but not anti-IgLON5 disease compared to healthy controls at all frequencies. However, patients with anti-IgLON5 disease could not be clearly distinguished from patients with PSP concerning SPEM at the frequencies studied. IV) We found spontaneous nystagmus and gaze evoked nystagmus in all our patients suffering from anti-IgLON5 disease but only in 50% of our patients with PSP. V) Clinically, all patients with PSP - but only one patient with anti-IgLON5 disease - presented with supranuclear gaze palsy.

Our results confirm previous reports underlining reduced saccade peak velocity, saccade amplitude and impaired gain of SPEM in patients suffering from PSP-P and PSP-RS compared to healthy controls (19, 20). We could not detect impairment of semicircular-canal function examined at 0.32 Hz sinusoidal rotation in our patients with PSP (21). Square wave jerks have been described to occur in the early stage of PSP which was also observed in our cohort (22).

Histopathological differences between the two diseases allow correlations with our results of ocular motor abnormalities. In contrast to anti-IgLON5 disease, PSP-P and PSP-RS patients more frequently exhibit tau pathologies in thalamic regions and basal ganglia (4, 23). In the context of later disease onset and shorter disease duration in PSP-RS compared to PSP-P the former harbors more extensive and severe tau pathology in certain limbic and brainstem areas particularly in the midbrain tegmentum, pontine basis, dentate nucleus and cerebellar white matter (24, 25). Cerebellar regions, i.e. fastigial nuclei, but also mesencephalic superior colliculi are affected to a lesser extent in anti-IgLON5 disease compared to PSP, which may explain the absence of SWJ and better saccade parameters in these patients. Beside the cerebellar hypothesis of SWJ generation, it has recently been shown that cortical structures, especially the temporal cortex, are involved in SWJ generation. It was hypothesized that atrophy of temporal cortical structures may lead to overactivity of the superior colliculus, leading to increased activity of excitatory burst neurons and less input to omnipause neurons in the brainstem. In reference to neuropathological characteristics of anti-IgLON5 disease, in which subcortical pTau deposits were found, with isolated tau pathology in the temporal cortex only, this could also explain the presence of SWJ in patients with PSP in which tau pathology is found more prominently over the course of the disease (4, 23, 26). SWJ were observed in 9/10 patients with PSP, but in none of our patients with anti-IgLON5 disease. SWJ may occur in normal individuals and their frequency may increase with age (27, 28). There is evidence of a higher rate of SWJ occurring in patients with PSP compared to older normal subjects, although an exact distinction by means of SWJ frequency cannot be drawn (28, 29). In our cohort of patients with PSP the mean SWJ frequency was 40/min (28-48/min, min-max.). The most obvious finding from the clinician´s point of view was supranuclear gaze palsy in all patients suffering from PSP, whereas this was found only in a single patient with anti-IgLON5 disease, indicating involvement of mesencephalic structures. In consideration of previous reports supranuclear gaze palsy was found in up to 59% of patients suffering from anti-IgLON5 disease (1). A possible mesencephalic dysfunction, however, is supported by slowed velocities of the vertical saccades in all our anti-IgLON5 patients. Interestingly, horizontal saccade velocities of these patients did not differ significantly from controls, possibly indicating that pontine areas are less involved at the beginning of the disease. Brainstem symptoms in our cohort of patients with anti-IgLON5 disease indicated mainly involvement of lower brainstem structures. This is reflected by better performance in saccade parameters, absence of SWJ and supranuclear gaze palsy in 4/4 and 3/4 patients with anti-IgLON5 disease, respectively. Neuropathological findings showed extensive tau pathology in mesencephalic and pontine regions of patients with PSP, but less pronounced in anti-IgLON5 disease, thus supporting our clinical findings (23). We could not detect significant differences in saccade parameters between patients suffering from PSP-P and PSP-RS. A previous small sample study indicated differences in saccadic gain and velocity between patients suffering from PSP-P and PSP-RS especially concerning horizontal directions though results were not significant (19). Patients with PSP-RS experience vertical oculomotor dysfunction and frequent falls early in the disease course (7). With increasing duration of disease, both subtypes may show clinical similarities and thus can be best distinguished within the first 2 years of the disease (30–32). This may explain the similarities in saccade parameters between the groups (PSP-RS and PSP-P) in our small sample.

Our study has some limitations. The results of this study have to be interpreted in context of the retrospective study design and small sample size. We aimed to compare early VOG findings after diagnosis of patients suffering from anti-IgLON5 disease with patients suffering from PSP-RS and PSP-P. We tried to identify relevant differences in ocular motor function between these patient groups that might be disease-specific and, thus, supportive for early diagnosis. The median interval from symptom onset to VOG examination was 6.5 years in anti-IgLON5 disease *vs.* 2 years in PSP-RS and PSP-P. Due to the chronic progressive course of both diseases, different disease stages are possible. It is noteworthy that all patients with anti-IgLON5 disease fulfilled at least the diagnostic criteria suggestive of PSP (2 suggestive of PSP/2 probable PSP) at the time of VOG and 2/4 patients with anti-IgLON5 disease presented with gait instability and ataxia but without typical parkinsonian features. Only one of those patients presented supranuclear gaze palsy. VOG may exhibit an additional value in differentiating between PSP and anti-IgLON5 disease which was indicated by our results, foremost concerning saccade accuracy and velocity and the presence of saccadic intrusions. The use of VOG as diagnostic, but also predictive or prognostic biomarker, could be addressed by further studies with larger sample sizes and longitudinal analysis of VOG findings, focusing on patients with anti-IglON5 disease without parkinsonian features but developing a PSP like phenotype over the course. As an additional vestibular finding, VORs was significantly reduced in patients with PSP, but not in anti-IgLON5 disease, compared to controls. To complete the vestibular examination, the application of lower and higher frequencies through caloric testing or video HIT would be useful. In this context we have to mention that the use of Video-HIT examination might have led to different results as recently described in an anecdotal report of bilateral vestibulopathy in a patient with anti-IgLON5 disease (33). Regarding to the small sample size and the pilot character of this study we did not aim to conduct any sensitivity-specificity analysis or define cut-off values especially for saccade parameters, but this could be approached by future studies. Finally, we cannot rule out that cognitive function may have influenced saccade latency (IgLON5 median Mini Mental Status Examination Score (MMSE): 26 points., min.-max. 21-28.; PSP median MMSE: 28.5 points; min.-max. 19-30).

Our study shows that ocular motor function in patients with anti-IgLON5 differs from those of patients with PSP, foremost concerning saccade velocity and accuracy, the presence of SWJ and the clinical finding of supranuclear gaze palsy, which was only found in a single patient with anti-IgLON5 disease. The use of VOG as a complementary neurophysiological tool for neurological assessment may assist in differentiating between patients with PSP and patients with anti-IgLON5 disease. Anti-IgLON5 antibodies are the main diagnostic biomarker but the assessment of ocular motor function, particularly in patients with suspected PSP and absence of typical Parkinson’s features, may prompt for anti-IgLON5 antibody testing and support accurate diagnosis. Longitudinal studies of ocular motor function in these patients may provide more distinct results and may highlight differences between the two patient groups more clearly. Furthermore, future studies should aim to determine to what extent ocular motor parameters could be used as diagnostic and monitoring biomarkers in anti-IgLON5 disease.

## Data Availability Statement

The original contributions presented in the study are included in the article/**Supplementary Material**. Further inquiries can be directed to the corresponding authors.

## Ethics Statement

The studies involving human participants were reviewed and approved by Ethikkommission der Medizinischen Universität Wien. Written informed consent for participation was not required for this study in accordance with the national legislation and the institutional requirements.

## Author Contributions

SM had full access to all of the data in the study and takes responsibility for the integrity of the data and the accuracy of the data analysis. Concept and design: SM, PR, GW, and IM. Acquisition, analysis, or interpretation of data: All authors. Drafting of the manuscript: SM, PR, GW, and IM. Critical revision of the manuscript for important intellectual content: All authors. Statistical analysis: SM. All authors contributed to the article and approved the submitted version.

## Conflict of Interest

The authors declare that the research was conducted in the absence of any commercial or financial relationships that could be construed as a potential conflict of interest.

## Publisher’s Note

All claims expressed in this article are solely those of the authors and do not necessarily represent those of their affiliated organizations, or those of the publisher, the editors and the reviewers. Any product that may be evaluated in this article, or claim that may be made by its manufacturer, is not guaranteed or endorsed by the publisher.

## Supplementary Material

Supplementary Figure 1 VOR Suppression

Supplementary Table 1 Saccade accuracy.

Supplementary Table 2 Saccade velocity

Supplementary Table 3 Saccade latency

## Abbreviations

CON, Controls; MDS, Movement Disorder Society; OCB, Oligoclonal bands; PSP, Progressive supranuclear palsy; SPEM, smooth pursuit eye movements; SWJ, Square wave jerks; VOG, Video-oculography; VOR, Vestibulo-ocular reflex.
