# Supplementary figures and images for: Ocular Motor Abnormalities in Anti-IgLON5 Disease
Source: Front Immunol. 2021 Sep 30;12:753856. doi: 10.3389/fimmu.2021.753856 (PMC8514941; doi:10.3389/fimmu.2021.753856)

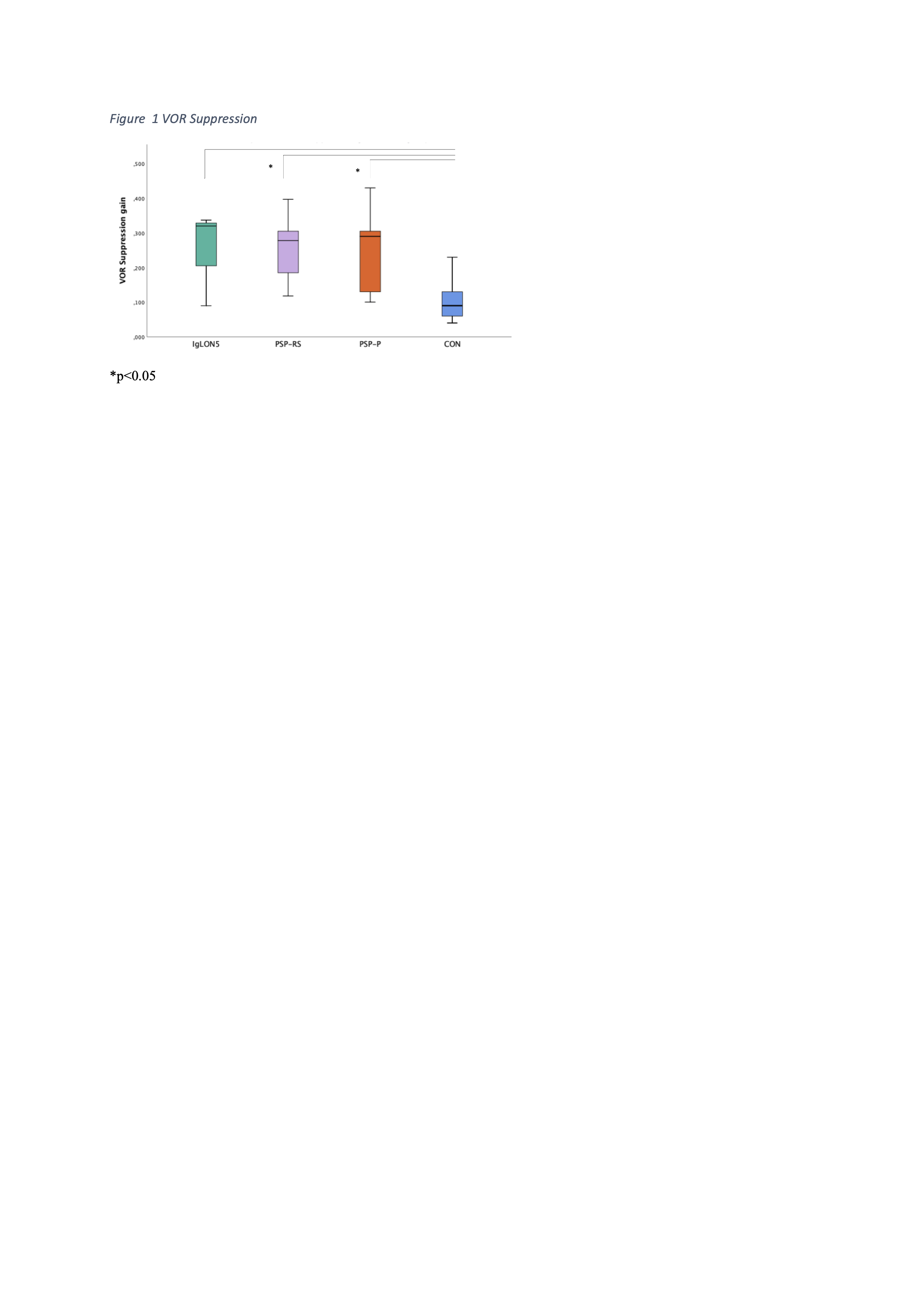

Supplement: Supplementary Figure 1 — VOR Suppression [file Image_1.tiff]
