# Supplementary material for: Ocular Motor Abnormalities in Anti-IgLON5 Disease
Source: Front Immunol. 2021 Sep 30;12:753856. doi: 10.3389/fimmu.2021.753856 (PMC8514941; doi:10.3389/fimmu.2021.753856)
Supplement: Supplementary Table 1 — Saccade accuracy. [file Table_1.docx]

Table 1 Saccade accuracy

| Saccade type | Patient Group | Median (%) | IQR (%) | Min.-Max.(%) | Intergroup comparison |
| --- | --- | --- | --- | --- | --- |
| Small horizontal | Anti-IgLON5 | 97 | 22.3 | 49-126 | IgLON5 vs. PSP-RS* |
|  | PSP-RS | 76.5 | 43 | 20-134 | IgLON5 vs. PSP-P* |
|  | PSP-P | 83.5 | 21.3 | 28-150 | PSP-RS vs. PSP-P |
|  | CON | 98 | 12 | 61-130 | CON vs. PSP-RS* |
|  |  |  |  |  | CON vs. PSP-P* |
|  |  |  |  |  | CON vs. IgLON5 |
| Large horizontal | Anti-IgLON5 | 93.5 | 13.75 | 82-109 | IgLON5 vs. PSP-RS* |
|  | PSP-RS | 65 | 24 | 24-100 | IgLON5 vs. PSP-P* |
|  | PSP-P | 76.5 | 25.5 | 36-101 | PSP-RS vs. PSP-P |
|  | CON | 92 | 10 | 44-110 | CON vs. PSP-RS* |
|  |  |  |  |  | CON vs. PSP-P* |
|  |  |  |  |  | CON vs. IgLON5* |
| Small vertical | Anti-IgLON5 | 95.5 | 22.5 | 47-155 | IgLON5 vs. PSP-RS* |
|  | PSP-RS | 71 | 35 | 25-206 | IgLON5 vs. PSP-P* |
|  | PSP-P | 72.5 | 32.3 | 9-137 | PSP-RS vs. PSP-P |
|  | CON | 98 | 17.5 | 69-131 | CON vs. PSP-RS* |
|  |  |  |  |  | CON vs. PSP-P* |
|  |  |  |  |  | CON vs. IgLON5 |
| Large vertical | Anti-IgLON5 | 95.5 | 22.5 | 47-155 | IgLON5 vs. PSP-RS* |
|  | PSP-RS | 71 | 35 | 25-206 | IgLON5 vs. PSP-P* |
|  | PSP-P | 72.5 | 32.3 | 9-137 | PSP-RS vs. PSP-P |
|  | CON | 92 | 16 | 63-127 | CON vs. PSP-RS* |
|  |  |  |  |  | CON vs. PSP-P* |
|  |  |  |  |  | CON vs. IgLON5 |

* p< 0.05 (Bonferroni correction)
