# Supplementary material for: Ocular Motor Abnormalities in Anti-IgLON5 Disease
Source: Front Immunol. 2021 Sep 30;12:753856. doi: 10.3389/fimmu.2021.753856 (PMC8514941; doi:10.3389/fimmu.2021.753856)
Supplement: Supplementary Table 2 — Saccade velocity [file Table_2.docx]

Table 2 Saccade velocity

| Saccade type | Patient Group | Median (deg/sec) | IQR (deg/sec) | Min.-Max.(deg/sec) | Intergroup comparison |
| --- | --- | --- | --- | --- | --- |
| Small horizontal | Anti-IgLON5 | 220 | 99.6 | 87.1-364 | IgLON5 vs. PSP-RS* |
|  | PSP-RS | 122.4 | 99.1 | 48.9-392.5 | IgLON5 vs. PSP-P* |
|  | PSP-P | 164.9 | 74 | 69.9-400.9 | PSP-RS vs. PSP-P |
|  | CON | 235.7 | 87.7 | 49.5-525.9 | CON vs. PSP-RS* |
|  |  |  |  |  | CON vs. PSP-P* |
|  |  |  |  |  | CON vs. IgLON5 |
| Large horizontal | Anti-IgLON5 | 358.8 | 86.9 | 221-466.6 | IgLON5 vs. PSP-RS* |
|  | PSP-RS | 227.6 | 101.7 | 82.3-600 | IgLON5 vs. PSP-P* |
|  | PSP-P | 255.8 | 144.3 | 115.8-500.8 | PSP-RS vs. PSP-P |
|  | CON | 378.2 | 94.6 | 182.5-600 | CON vs. PSP-RS* |
|  |  |  |  |  | CON vs. PSP-P* |
|  |  |  |  |  | CON vs. IgLON5 |
| Small vertical | Anti-IgLON5 | 179.7 | 63.7 | 60-301 | IgLON5 vs. PSP-RS* |
|  | PSP-RS | 49 | 59.4 | 12.9-344.2 | IgLON5 vs. PSP-P* |
|  | PSP-P | 93 | 61 | 6.4-289.9 | PSP-RS vs. PSP-P |
|  | CON | 242.4 | 70.1 | 99.5-438.5 | CON vs. PSP-RS* |
|  |  |  |  |  | CON vs. PSP-P* |
|  |  |  |  |  | CON vs. IgLON5* |
| Large vertical | Anti-IgLON5 | 298.2 | 70.1 | 165.7-402.5 | IgLON5 vs. PSP-RS |
|  | PSP-RS | 226.2 | 106.8 | 120.6-314.1 | IgLON5 vs. PSP-P |
|  | PSP-P | 173.7 | 151.2 | 74.3-340.5 | PSP-RS vs. PSP-P |
|  | CON | 362.2 | 92.8 | 214.4-543.2 | CON vs. PSP-RS* |
|  |  |  |  |  | CON vs. PSP-P* |
|  |  |  |  |  | CON vs. IgLON5* |

* p<0.05 (Bonferroni correction)
