# Supplementary material for: Ocular Motor Abnormalities in Anti-IgLON5 Disease
Source: Front Immunol. 2021 Sep 30;12:753856. doi: 10.3389/fimmu.2021.753856 (PMC8514941; doi:10.3389/fimmu.2021.753856)
Supplement: Supplementary Table 3 — Saccade latency [file Table_3.docx]

Table 3 Saccade latency

| Saccade type | Patient Group | Median (ms) | IQR (ms) | Min.-Max.(ms) | Intergroup comparison |
| --- | --- | --- | --- | --- | --- |
| Small horizontal | Anti-IgLON5 | 233.3 | 58.3 | 150-400 | IgLON5 vs. PSP-RS |
|  | PSP-RS | 250 | 62.5 | 16.7-633.3 | IgLON5 vs. PSP-P |
|  | PSP-P | 250 | 66.7 | 133.3-416-7 | PSP-RS vs. PSP-P |
|  | CON | 216.7 | 66.7 | 150-333.3 | CON vs. PSP-RS* |
|  |  |  |  |  | CON vs. PSP-P* |
|  |  |  |  |  | CON vs. IgLON5 |
| Large horizontal | Anti-IgLON5 | 233.3 | 66.6 | 183.3-316.7 | IgLON5 vs. PSP-RS* |
|  | PSP-RS | 266.7 | 116.7 | 200-483.3 | IgLON5 vs. PSP-P |
|  | PSP-P | 250 | 50 | 183.3-383.3 | PSP-RS vs. PSP-P |
|  | CON | 216.7 | 50 | 150-316.7 | CON vs. PSP-RS* |
|  |  |  |  |  | CON vs. PSP-P* |
|  |  |  |  |  | CON vs. IgLON5* |
| Small vertical | Anti-IgLON5 | 283.3 | 54.2 | 183.3-466.7 | IgLON5 vs. PSP-RS* |
|  | PSP-RS | 450 | 200 | 50-683.3 | IgLON5 vs. PSP-P* |
|  | PSP-P | 400 | 237.5 | 183.3-683.3 | PSP-RS vs. PSP-P |
|  | CON | 216.7 | 54.2 | 150-333.3 | CON vs. PSP-RS* |
|  |  |  |  |  | CON vs. PSP-P* |
|  |  |  |  |  | CON vs. IgLON5* |
| Large vertical | Anti-IgLON5 | 258.4 | 58.4 | 216.7-400 | IgLON5 vs. PSP-RS |
|  | PSP-RS | 283.3 | 83.3 | 216.7-366.7 | IgLON5 vs. PSP-P |
|  | PSP-P | 283.3 | 87.5 | 233.3-433.3 | PSP-RS vs. PSP-P |
|  | CON | 233.3 | 66.7 | 150-350 | CON vs. PSP-RS* |
|  |  |  |  |  | CON vs. PSP-P* |
|  |  |  |  |  | CON vs. IgLON5* |

*p<0.05 (Bonferroni correction)
